# Severest Anæmias: Their Infective Nature, Diagnosis and Treatment

**Published:** 1910-09

**Authors:** 


### Reviews of Books.

**Severest Anæmias: their Infective Nature, Diagnosis and Treatment.** By WILLIAM HUNTER, M.D. Vol. I. Pp. xv, 226. London: Macmillan & Co. 1909.

Hæmatologists have evolved a remarkable terminology for their particular branch of medical science; it tends to confuse the average reader. Whether the bold stand taken by the writer of *Severest Anæmias* will effect any simplification in the descriptions of blood changes remains to be seen, but it will go a long way towards convincing the general worker that the many differentiations of the cellular elements of the blood lead to no tangible end. When an observer like Hunter clears away the mists surrounding the earlier conceptions of "pernicious anæmia," tells how the accurate descriptions of the first English writers on the disease were made less clear by subsequent writers, and replaces the hypothesis of degradation of blood cells to embryonic types by attributing these changes to an infectious origin, we feel that at last the way opens for clearer thought. Rightly or wrongly, this conception has gained considerable hold already, and there are many who unhesitatingly side with Hunter in regarding degeneration and atrophic changes in the mucous membrane of the tongue, stomach and intestines as the all-sufficient factor in the ætiology of severe anæmia. The dietetic and medicinal treatment of the alimentary tract in many cases tends to the cure and arrest of the disease, and the consequent disappearance or diminution of the anæmia.

Perhaps, however, the matter is not such a simple one. While it must be admitted that infections of an alimentary origin may play a part in the production of anæmia, we are not warranted in ascribing all severe anæmias to this cause.

Nevertheless this work of Dr. Hunter is exceedingly valuable. It tends to the restoration of clinical observation, and points away from the absolute necessity of laboratory diagnosis in severe anæmia. Much of it has appeared before in medical periodicals, much of it is a review of the papers on pernicious anæmia published during the last forty years, but it is valuable to have all the information within the pages of one volume, and we therefore recommend the purchase of this book to those who are interested in the subject.

